# Acupuncture combined with metformin versus metformin alone to improve pregnancy rate in polycystic ovary syndrome: A systematic review and meta-analysis

**DOI:** 10.3389/fendo.2022.978280

**Published:** 2022-08-29

**Authors:** Xin Chen, Ying Lan, Lijie Yang, Yang Liu, Hongyu Li, Xinyun Zhu, Yuemeng Zhao, Caiyi Long, Mengjing Wang, Qingling Xie, Zhao Li, Jie Wu

**Affiliations:** ^1^ College of Acupuncture and Tuina, Chengdu University of Traditional Chinese Medicine, Chengdu, China; ^2^ Hospital of Chengdu University of Traditional Chinese Medicine, Chengdu, China; ^3^ People’s Hospital of Leshan, Leshan, China; ^4^ Clinical Medical College, Chengdu University of Traditional Chinese Medicine, Chengdu, China

**Keywords:** acupuncture, metformin, polycystic ovary syndrome, pregnancy rate, insulin resistance, systematic review, meta-analysis

## Abstract

**Objective:**

The aim of this study was to evaluate the comparison between acupuncture combined with metformin versus metformin alone in improving the pregnancy rate of people with polycystic ovary syndrome (PCOS).

**Methods:**

A literature search of eight databases resulted in nine randomized controlled trials (RCTs) that assessed the effect of acupuncture combined with metformin on pregnancy rate in PCOS patients compared with metformin alone. Subsequently, data extraction and analysis were conducted to evaluate the quality and risk of bias of the methodological design of the study, and meta-analysis was conducted on the RCT data.

**Results:**

Nine RCTs and 1,159 women were included. Acupuncture can improve pregnancy rate. It was analyzed according to the diagnostic criteria of PCOS [*Z* = 2.72, *p* = 0.007, relative risk (RR) 1.31, 95% CI 1.08 to 1.60, *p* = 0.15, *I* ^2^ = 41%]. Analysis was performed according to different diagnostic criteria of pregnancy (*Z* = 3.22, *p* = 0.001, RR 1.35, 95% CI 1.13 to 1.63, *p* = 0.12, *I* ^2^ = 42%). Acupuncture can improve ovulation rate. Subgroup analysis was performed according to the number of ovulation patients (*Z* = 2.67, *p* = 0.008, RR 1.31, 95% CI 1.07 to 1.59, *p* = 0.04, *I* ^2^ = 63%) and ovulation cycle (*Z* = 3.57; *p* = 0.0004, RR 1.18, 95% CI 1.08 to 1.29, *p* = 0.57, *I* ^2^ = 0%). Statistical analysis also showed that acupuncture combined with metformin could improve homeostatic model assessment of insulin resistance (HOMA-IR) [mean difference (MD) −0.68, 95% CI −1.01 to −0.35, *p* = 0.003, *I* ^2^ = 83%].

**Conclusions:**

Based on the results of this study, compared with metformin alone, acupuncture combined with metformin has a positive effect on pregnancy rate, ovulation rate, and insulin resistance in PCOS. However, due to the limitations regarding the number and quality of the included studies, the above conclusions need to be verified by further high-quality studies.

**Systematic Review Registration:**

https://www.crd.york.ac.uk/PROSPERO/#myprospero.

## Introduction

Polycystic ovary syndrome (PCOS) is the most common hormonal disorder in women and is also one of the most common factors that cause infertility (1). With the opening of the three-child policy in China, the reproductive needs of women of childbearing age are increasingly urgent, but the incidence of PCOS in these women is as high as 9% to 18% (2), which has a great impact on pregnancy. Studies have shown that insulin resistance (IR) is a key feature of the pathophysiology of PCOS (3), with 85% of patients being affected by IR. IR disrupts the follicular environment (4) by leading to hyperandrogenemia (5), affecting follicular development and ovulation, which is not conducive to pregnancy. In addition, people with obesity account for 35%–60% (6) of the population with PCOS, which is closely related to IR and the pathological mechanism of PCOS (7). Weight gain has been shown to further aggravate IR (8, 9). The above factors affect women’s health.

Acupuncture has become more and more popular in the world as a complementary and alternative therapy for infertility. In 2010, a study in the United States showed that 29% of patients used complementary and alternative drugs with the aim to treat infertility, of whom 22% chose acupuncture (10). In China, traditional medicine is even more popular. A large number of clinical and animal experiments have shown that acupuncture has significant effects in the treatment of infertility and anovulation caused by PCOS, including improving clinical pregnancy rate, ovulation rate, live birth rate, insulin resistance, menstruation, hormone levels, follicular development, and hyperandrogenaemia, and regulating the secretory function of hypothalamic pituitary ovarian axis (HPOA) (11–17) but it may also cause subcutaneous bleeding or pain and other mild adverse reactions. Studies have shown that metformin is one of the most important drugs for reducing insulin resistance in PCOS patients (18). Such drugs have been shown to improve clinical pregnancy rate and ovulation rate, and have positive effects on hyperinsulinemia and ovarian androgen hypersecretion.

There are no detailed and systematic methodological evaluations and data consolidations between acupuncture combined with metformin versus metformin alone. The main objective of this study was to conduct a systematic review and meta-analysis comparing whether acupuncture combined with metformin can further improve pregnancy rates compared to metformin alone, thus providing a more effective treatment for this population.

## Methods

This study was conducted according to Preferred Reporting Items for Systematic Reviews and Meta-analyses (PRISMA) (19). This study was listed on the Prospective Register of Systematic Reviews (PROSPERO) on 15 February 2022, with registration number: CRD42022302940.

### Data sources

In this study, the following eight databases [PubMed, Embase, Cochrane Central Register of Controlled Trials (CENTRAL), Web of Science (SCI), Chinese Biomedical Database (CBM), Chinese National Knowledge Infrastructure (CNKI), Wan Fang Data Knowledge Service Platform, and VIP Journal Integration Platform (VIP)] were searched from the database construction to 14 April 2022, including four English databases and four Chinese databases. At the same time, to retrieve Chinese Clinical Trial Register, Clinical Trials.gov, relevant articles in the reference lists were also collected. The retrieval was carried out by the combination of medical subject headings (MeSH). Keywords searched in PubMed include “Acupuncture” and “Metformin” and “Polycystic Ovary Syndrome” and “Infertility” and “Randomized Controlled Trial”. See appendix for detailed search strategies.

### Study selection and data extraction

Two researchers (YL and HYL) independently screened the retrieved articles, read the titles and abstracts, and then excluded the repeated studies and irrelevant articles. According to the inclusion criteria and exclusion criteria, eligible studies were identified, data were extracted and cross-checked, and any ambiguity was resolved through discussion and consensus. If no consensus was reached, a third researcher (QLX) was asked to adjudicate. Any literature that is removed will be recorded. The experimental groups received acupuncture (acupuncture, moxibustion, electroacupuncture, acupoint embedding therapy, acupoint injection, acupuncture ear, warm needling, fire-needle, or floating needle) combined with metformin; the control groups were treated with metformin alone. Inclusion criteria were as follows: (a) subjects were diagnosed with PCOS; (b) the treatment group used acupuncture combined with metformin, while the control group used only metformin; and (c) the study type was a randomized controlled trial. Exclusion criteria were as follows: (a) subjects were treated with drugs other than metformin; (b) the use of traditional Chinese medicine; (c) the study was conducted on animals; and (d) the study was not reported in Chinese or English. This study was divided into two groups: the acupuncture combined with metformin group and the metformin alone group. Data were extracted independently by two researchers (YL and HYL), and checked by another researcher (XC) to extract information that might be related to the research results, as follows: First author, year of publication, number of participants, age, infertility duration, treatment duration, interventions, diagnostic criteria, outcome indicators, side effects and adverse events, and other information. The results were recorded in an Excel spreadsheet (**Table 1**).

**Table 1:** Characteristics of the studies included in this systematic review (acupuncture + metformin vs. metformin).

No.	Study, publication year(country)	No. of patients(O/A)	Age: mean±SD or range(years)	Duration of infertility: mean±SD or range (years)	Intervention	Control	Period of treatment	Diagnostic criteria	Side effects and adverse events	Type of outcomes
**1**	**Li YC,2021 (China)21**	**I:57/57 C:57/57**	**I:31.42±3.22(20-40) C:31.48±3.25(21-39)**	**NR**	**Acupuncture+Metformin**	**Metformin**	**3 months**	**2018 "Guidelines for Diagnosis and Treatment of Polycystic Ovarian Syndrome" in China**	**NR**	**pregnancy, ovulation, TCM symptom score,FPG,FINS,HOMA-IR,Blood fat.**
**2**	**Wang JY,2020 (China) 22**	**I:30/30 C:30/30**	**I:26.43±3.52(22-38) C:26.23±3.48(21-38)**	**NR**	**Acupuncture+Metformin**	**Metformin**	**3 months**	**NR**	**NR**	**pregnancy,Sex hormone level,FPG,HOMA-IR,BMI,etc.**
**3**	**Li L,2014 (China) 23**	**I:53/53 C:51/51**	**I:27.10±2.50(22-40) C:25.20±1.80(21-38)**	**I:3.6(2.0-7.5) C:3.3(2.0-7.2)**	**Acupuncture+Metformin**	**Metformin**	**6 months**	**Rotterdam**	**Gastrointestinal reaction**	**Sex hormone level,BBT,B-scan ultrasonography,BMI,WHR,Ferriman-Gallway,HOMA-IR,FPG,FINS,OGTT,ST,FBG,etc.**
**4**	**Zhai ZY,2017 (China) 24**	**I:40/40 C:40/40**	**I:23.70±2.20(20-29) C:23.10±1.90(20-28)**	**I:4.40±1.20(2-7) C:4.10±1.40(2-7)**	**Acupuncture+Metformin**	**Metformin**	**3 months**	**Rotterdam**	**Gastrointestinal reaction,Menstrual abnormalities**	**ovulation,BMI,WHR,Sex hormone level,FPG,FINS, APN.**
**5**	**Peng XY,2020 (China) 25**	**I:30/30 C:30/30**	**I:28.42±1.32(22-32) C:28.32±1.35(22-33)**	**I:2.02±0.12(0.6-4) C:2.06 ± 0.11(0.6-4)**	**Acupuncture+Metformin**	**Metformin**	**6 months**	**2018 "Guidelines for Diagnosis and Treatment of Polycystic Ovarian Syndrome" in China**	**NR**	**pregnancy,ovulation,Sex hormone level, B-scan ultrasonography.**
**6**	**Li SS,2015 (China) 26**	**I:75/75 C:75/75**	**I:25.10±2.30(23-34) C:24.10±2.20(21-33)**	**I:3.2(2-4) C:3.3(2-5)**	**Acupuncture+Metformin**	**Metformin**	**6 months**	**NR**	**NR**	**pregnancy,ovulation,Sex hormone level, B-scan ultrasonography,HOMA-IR,BMI,Ferriman-Gallway,OGTT,ST.**
**7**	**Tang J,2017 (China) 27**	**I:52/52 C:52/52**	**I:28.53± 6.62(22-42) C:28.26±6.70(21-40)**	**I:3.72±1.38(1-7) C:3.41±1.52(1-8)**	**Acupuncture+Metformin**	**Metformin**	**I:3 menstrual cycles C:3 months**	**Rotterdam**	**NR**	**pregnancy,ovulation,Total effects,BMI,FPG,Sex hormone level,FINS.**
**8**	**Zhang ZL,2016 (China) 28**	**I:50/50 C:50/50**	**I:26.30± 4.40(22-38) C:27.2±4.1(23-37)**	**I:3.2±0.6(2-7) C:3.1± 0.5(2-6)**	**Acupuncture+Metformin**	**Metformin**	**6 months**	**Rotterdam**	**NR**	**BMI,WHR,B-scan ultrasonography,Sex hormone level,Ferriman-Gallway**
**9**	**Liu YE, 2018 (China)29**	**I:138/138 C:140/140**	**I:30.00±7.00(23-37) C:31.20±8.10(23-39)**	**I:2.35±1.65(0.7-4.0) C:2.51± 1.83(0.7-4.3)**	**Acupuncture+Metformin**	**Metformin**	**2 months**	**Rotterdam**	**NR**	**pregnancy,ovulation,Sex hormone level,AMH,BMI,menstrual cycle,etc.**

AMH, anti-Müllerian hormone; APN, Adiponectin;BBT, basal body temperature; BMI,Body Mass Index; C, control; FINS,Fasting insulin; FPG, fasting plasma glucose; HOMA-IR, Homeostatic model assessment of insulin resistance; I, intervention; NR, not reported; OGTT, oral glucose tolerance test; ST, insulin release test; TCM, Traditional Chinese Medicine; WHR, Waist-to-Hip Ratio.

### Risk of bias assessment

**Figure 1:**
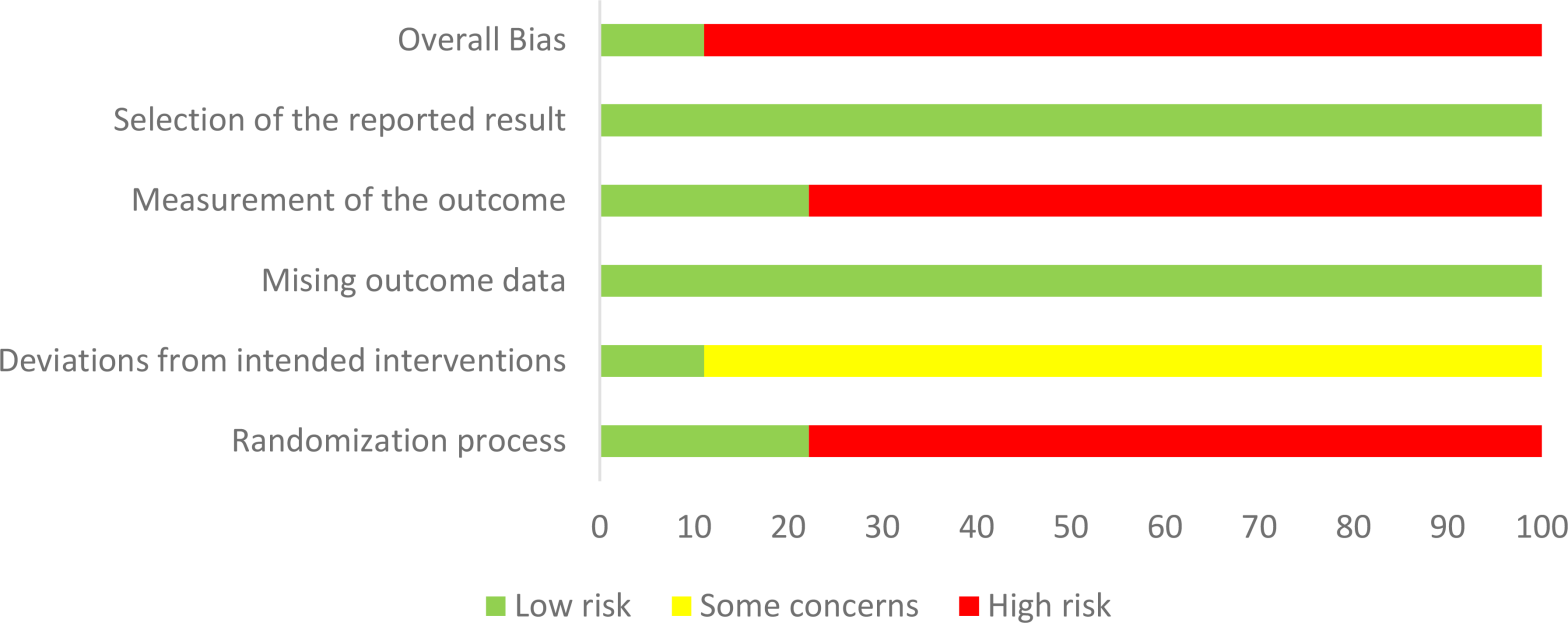
Risk of bias summary.

**Figure 2:**
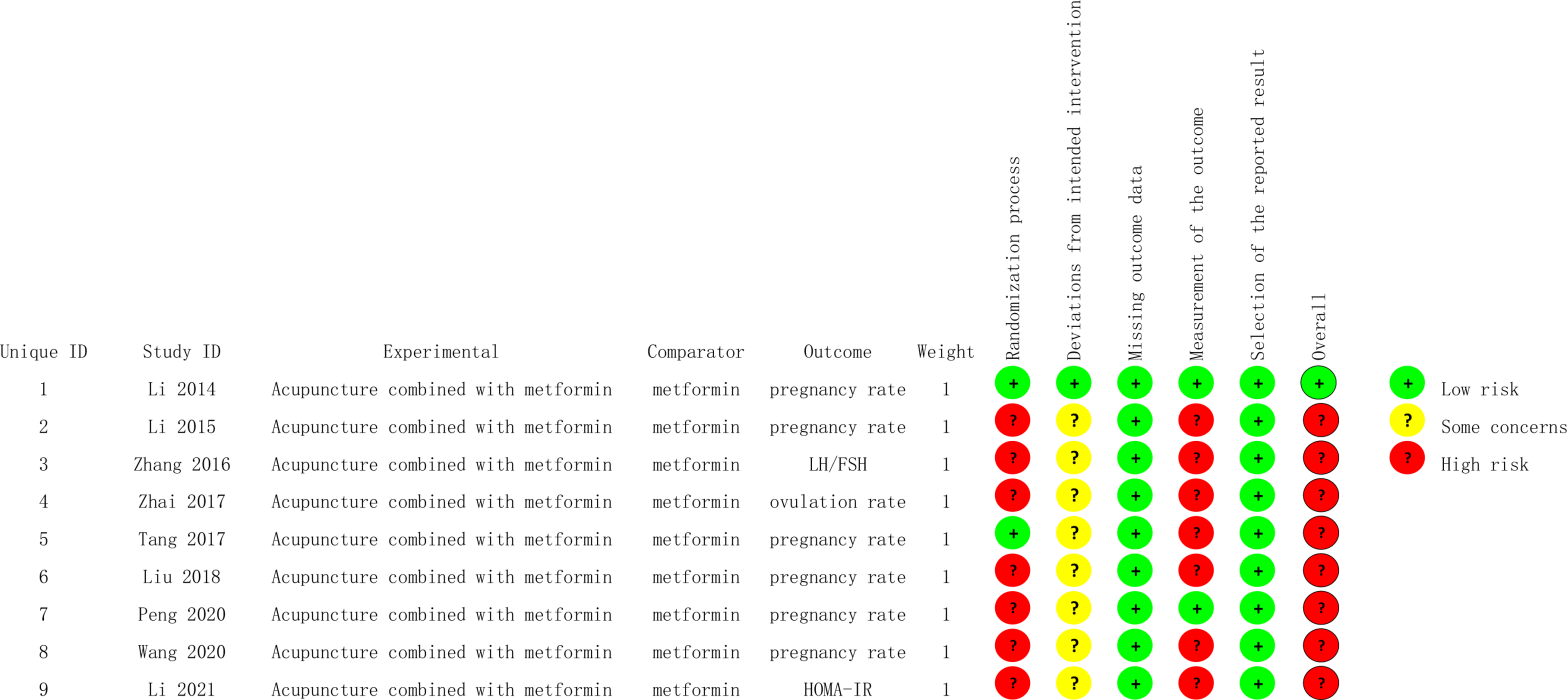
Risk of bias graph.

Cochrane RoB2.0 was used to evaluate risk of bias in the individual studies (20). The following six items were extracted from each of the RCTs for evaluation: (a) randomization process; (b) deviations from intended interventions; (c) missing outcome data; (d) measurement of the outcome; (e) selection of the reported result; and (f) overall. When the appropriate method is used and described appropriately and clearly, the study was considered to be low risk; otherwise, it was rated as high risk, or some concerns if the method could not be accurately judged. Two researchers (YL and HYL) independently assessed these factors and, if necessary, a third researcher (QLX) was consulted to resolve disagreements (**Figures 1**, **2**).

### Outcomes

Main outcome measures: pregnancy rate: positive morning urine beta human chorionic gonadotropin (β-hCG) or blood β-hCG, basal body temperature (BBT) for more than 3 weeks, pregnancy sac detected by color ultrasonography, or fetal bud and heartbeat detected by colour ultrasonography at 7 weeks of gestation.

### Statistical analysis

RevMan5.3 software was used for statistical analysis. Relative risk (RR) and its 95% confidence interval (CI) were used for dichotomous variables, MD or standardized mean difference (SMD) were used for continuity variables, and 95% CI was given. *p* < 0.05 was considered as a statistically significant difference. According to the study of the object of study, observation group intervention, and control group, judging whether the concrete application of similar clinical outcome index, according to the result of the *I* ^2^ test to determine the statistical heterogeneity. *I* ^2^>50% is considered as high heterogeneity. The methods of subgroup analysis and sensitivity analysis were used, and the study adopts a random-effects model.

## Results

### Studies retrieved

**Figure 3:**
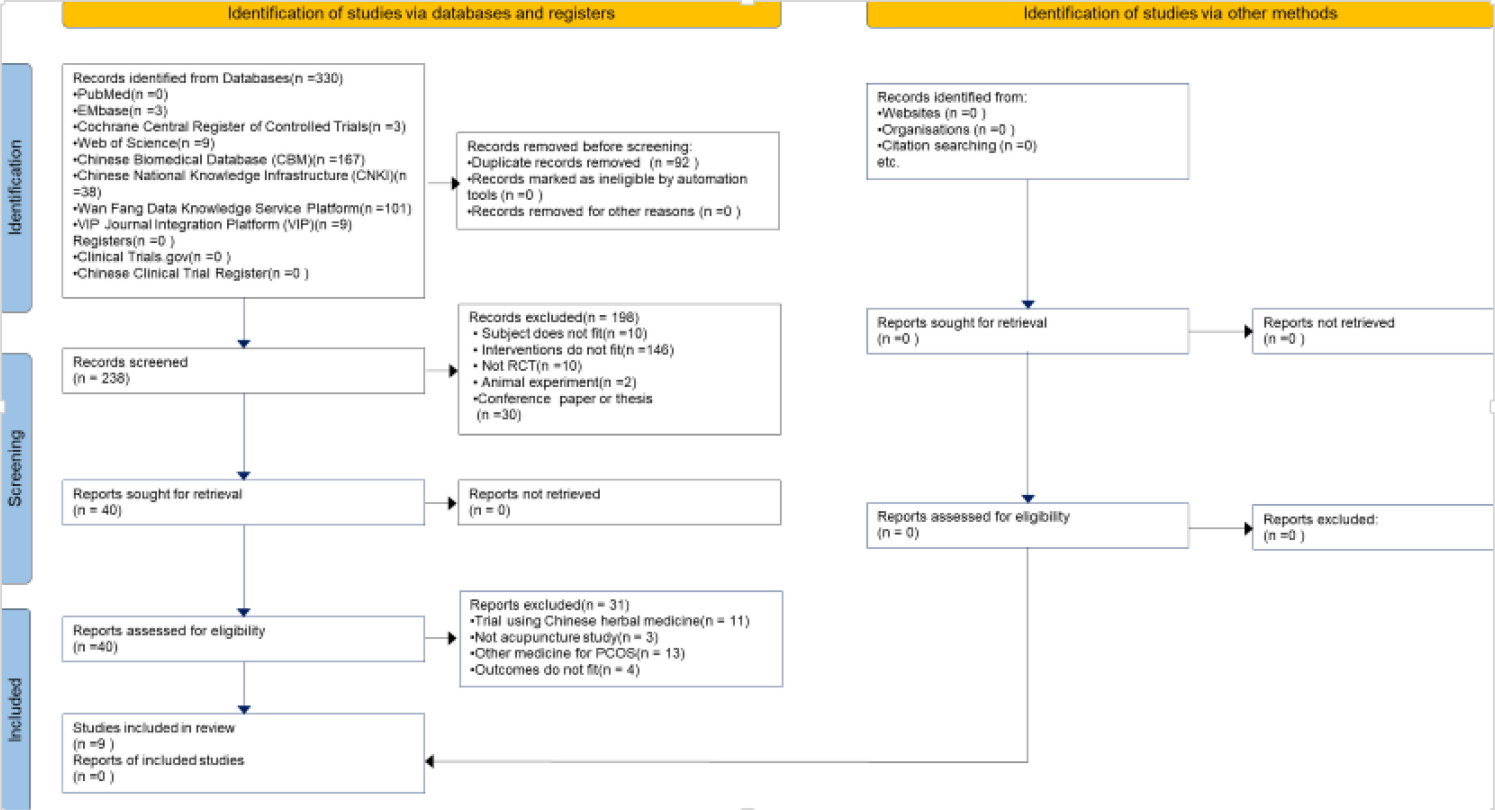
Flowchart of the study selection process.

In total, 330 related literatures were screened initially, and nine studies (21−29) ultimately met our inclusion criteria after the different rounds of screening. A total of 1,159 patients with PCOS who received acupuncture or acupuncture combined with metformin were identified from nine randomized controlled trials (**Figure 3**).

### Quality of the evidence: Summary of findings table

We used the GRADE method to present a “Summary of Findings” table. The quality of evidence for outcome measures (pregnancy rate, ovulation rate, and HOMA-IR) was evaluated for a subject review comparison (acupuncture combined with metformin vs. metformin alone). We used the GRADE criteria to assess the quality of evidence, study limitations, inconsistencies, inaccuracies, and publication bias. The two researchers (ZL and QLX) independently judged the quality of the evidence (high, moderate, low, or very low) and resolved differences through discussion **Table 2**.

**Table 2:** Summary of findings table.

Acupuncture+ Metformin compared to Metformin for PCOS								
**Patient or population:** patients with PCOS **Settings:** outpatients **Intervention:** Acupuncture+ Metformin **Comparison:** Metformin								
**Outcomes**	Illustrative comparative risks* (95% CI)			**Relative effect (95% CI)**	**No of Participants (studies)**	**Quality of the evidence (GRADE)**	**Comments**	
	Assumed risk		Corresponding risk					
	**Metformin**		**Acupuncture+ Metformin**					
**Pregnancy- B-ultrasound**	**Study population**			**RR 1.49** (1.07 to 2.07)	502 (4 studies)	⊕⊕⊝⊝ **low** ^1,2^		
	**563 per 1000**		**840 per 1000** (603 to 1000)	
	**Moderate**			
	**404 per 1000**		**602 per 1000** (432 to 836)	
**Pregnancy- Not B-ultrasound**	**Study population**			**RR 1.3** (1.02 to 1.65)	254 (2 studies)	⊕⊕⊝⊝ **low** ^1^		
	**429 per 1000**		**557 per 1000** (437 to 707)	
	**Moderate**			
	**407 per 1000**		**529 per 1000** (415 to 672)	
**Pregnancy- Rotterdam**	**Study population**			**RR 1.24** (1.06 to 1.45)	636 (4 studies)	⊕⊕⊝⊝ **low** ^1^		
	**547 per 1000**		**678 per 1000** (580 to 793)	
	**Moderate**			
	**414 per 1000**		**513 per 1000** (439 to 600)	
**Pregnancy- China**	**Study population**			**RR 2.14** (1.02 to 4.49)	60 (1 study)	⊕⊕⊝⊝ **low** ^1,3^		
	**233 per 1000**		**499 per 1000** (238 to 1000)	
	**Moderate**			
	**233 per 1000**		**499 per 1000** (238 to 1000)	
**Ovulation - number of ovulations**	**Study population**			**RR 1.31** (1.07 to 1.59)	532 (4 studies)	⊕⊕⊝⊝ **low** ^1,2^		
	**674 per 1000**		**883 per 1000** (721 to 1000)	
	**Moderate**			
	**649 per 1000**		**850 per 1000** (694 to 1000)	
**Ovulation - ovulation cycles**	**Study population**			**RR 1.18** (1.08 to 1.29)	683 (3 studies)	⊕⊕⊝⊝ **low** ^1^		
	**677 per 1000**		**798 per 1000** (731 to 873)	
	**Moderate**			
	**674 per 1000**		**795 per 1000** (728 to 869)	
**HOMA-IR**		The mean homa-ir in the intervention groups was **0.68 lower** (1.01 to 0.35 lower)			282 (3 studies)	⊕⊕⊝⊝ **low** ^1,3,4^		

*The basis for the assumed risk (e.g. the median control group risk across studies) is provided in footnotes. The corresponding risk (and its 95% confidence interval) is based on the assumed risk in the comparison group and the relative effect of the intervention (and its 95% CI). CI, Confidence interval; RR, Risk ratio; GRADE Working Group grades of evidence High quality, Further research is very unlikely to change our confidence in the estimate of effect. Moderate quality, Further research is likely to have an important impact on our confidence in the estimate of effect and may change the estimate. Low quality, Further research is very likely to have an important impact on our confidence in the estimate of effect and is likely to change the estimate. Very low quality, We are very uncertain about the estimate. ^1^Evidence downgraded by two levels for serious risk of bias, the majority of the RCTs have unclear or high risk of bias. ^2^Evidence downgraded by one level for serious inconsistency 50%<I2<75%. ^3^Evidence downgraded by one level for serious imprecision, low number of events (total number of events < 300). ^4^Evidence downgraded by two levels for serious inconsistency I2≥75%.

## Main results

### Pregnancy

**Figure 4:**
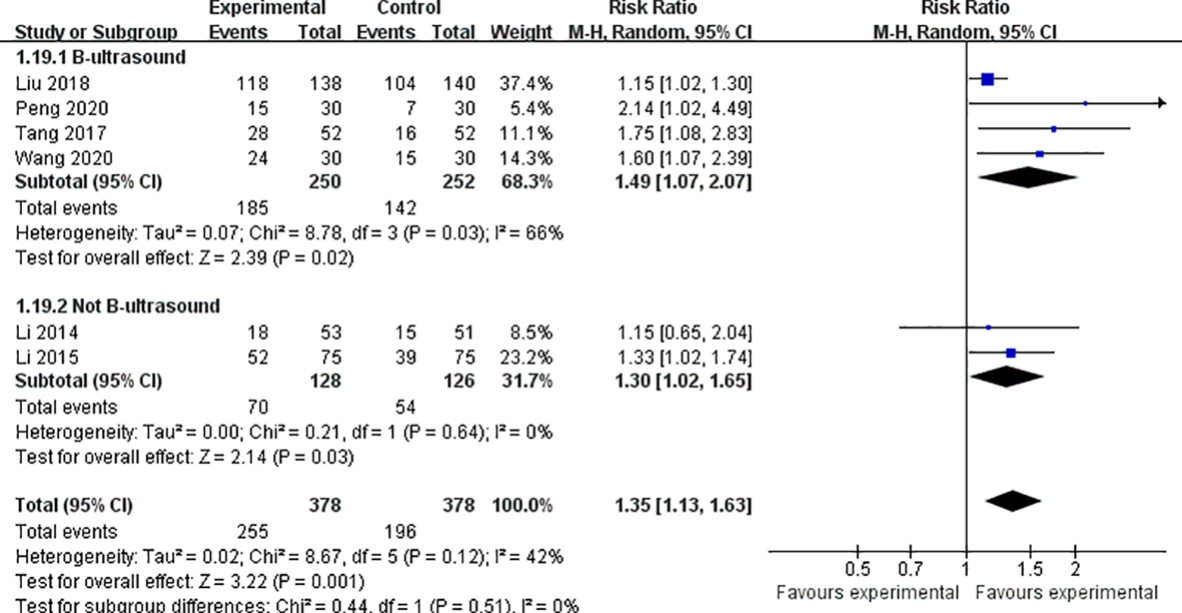
Forest plot of effects of acupuncture combined with metformin versus metformin alone on pregnancy rate (diagnostic criteria of pregnancy).

**Figure 5:**
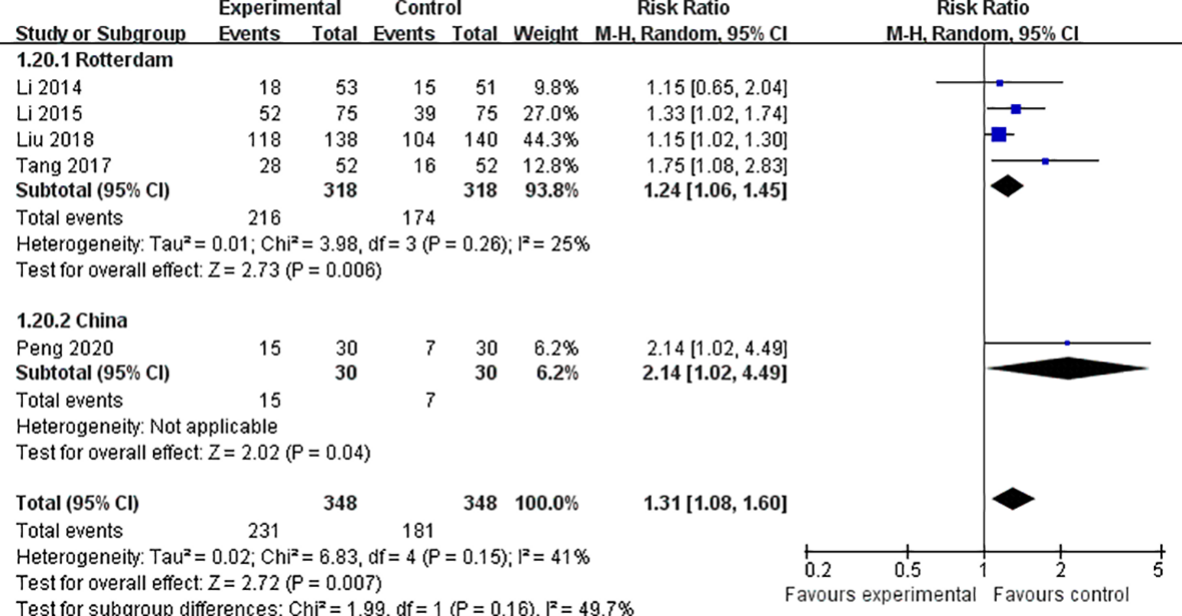
Forest plot of effects of acupuncture combined with metformin versus metformin alone on pregnancy rate (diagnostic criteria of PCOS).

Six studies of acupuncture combined with metformin reported pregnancy rates (22, 23, 25–27, 29). In terms of pregnancy rates, the results were statistically significantly different between the two groups (*Z* = 3.22, *p* = 0.001, RR 1.35, 95% CI 1.13 to 1.63, *p* = 0.12, *I* ^2^ = 42%), indicating that acupuncture combined with metformin was superior to metformin alone in improving pregnancy. We found that different diagnostic criteria were adopted in clinical studies on pregnancy rate; thus, we conducted subgroup analysis and used B-ultrasound for diagnosis (*Z* = 2.39, *p* = 0.02, RR 1.49, 95% CI 1.07 to 2.07, *p* = 0.03, *I* ^2^ = 66%). B-ultrasound was not used for the diagnostic group (*Z* = 2.14, *p* = 0.03, RR 1.30, 95% CI 1.02 to 1.65, *p* = 0.64, *I* ^2^ = 0%), indicating that the heterogeneity of the test was mainly derived from the criteria of pregnancy (**Figure 4**). Additionally, there are different diagnostic criteria for PCOS; when summarizing the characteristics, we found that there are two different sets of diagnostic criteria. The first is the Rotterdam standard, and the second is a set of PCOS diagnosis and treatment guidelines in China. We also performed subgroup analysis of the above two kinds of diagnostic criteria, finding that heterogeneity when using the Rotterdam criteria (*I* ^2^ = 25%) was significantly lower than the overall heterogeneity (*I* ^2^ = 41%) (**Figure 5**).

### Ovulation

Seven studies of acupuncture combined with metformin reported ovulation rates, calculated by number of ovulations in four studies (21, 24, 25, 29) and ovulation cycles in three studies (23, 26, 27). We analyzed these studies separately according to their different calculation methods.

**Figure 6:**
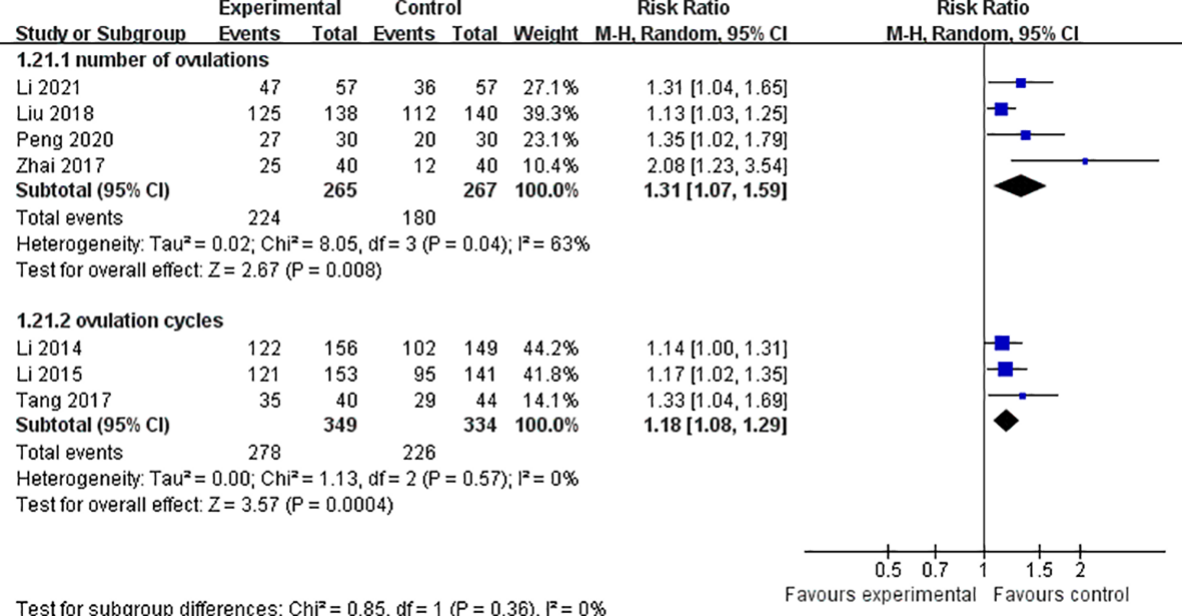
Forest plot of effects of acupuncture combined with metformin versus metformin alone on ovulation rate.

Calculated by the number of ovulation: 532 participants in these studies, 224 women in the trial group and 180 women in the control group ovulated. The results were statistically significant (*Z* = 2.67, *p* = 0.008, RR 1.31, 95% CI 1.07 to 1.59, *p* = 0.04, *I* ^2^ = 63%). Calculated according to ovulation cycle: 683 participants in these studies, cycle ovulation rates were recorded for 278 women in the trial group and 226 women in the control group. The results had low heterogeneity, and indicated that acupuncture combined with metformin had a positive effect on improving ovulation rate compared with metformin alone (*Z* = 3.57; *p* = 0.0004, RR 1.18, 95% CI 1.08 to 1.29, *p* = 0.57, *I* ^2^ = 0%) (**Figure 6**).

### Homa-Ir

**Figure 7:**
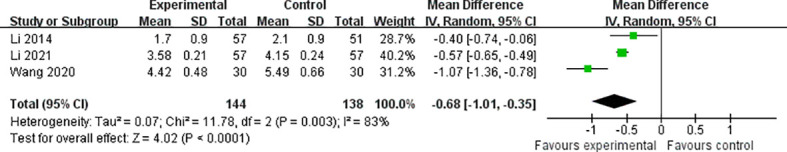
Forest plot of effects of acupuncture combined with metformin versus metformin alone on HOMA-IR.

HOMA-IR was reported in three studies of acupuncture combined with metformin (21–23) with a total of 282 participants, 144 in the experimental group and 138 in the control group. The results showed that the difference in HOMA-IR between the two groups was statistically significant (*Z* = 4.02; *p* < 0.0001), with high heterogeneity (MD −0.68, 95% CI −1.01 to −0.35, *p* = 0.003, *I* ^2^ = 83%). After sensitivity analysis (*p* = 0.34, *I* ^2^ = 0%), heterogeneity was derived from a research (22), and it was found that the sample size of the study was only 30 cases. Therefore, sample size may be the main source of heterogeneity (**Figure 7**).

### Side effects and adverse events

The incidence of side effects and adverse events was reported in two trials. A total of 21 patients treated with acupuncture combined with metformin had gastrointestinal reactions and 2 had menstrual abnormalities. After metformin alone treatment, a total of 23 patients had gastrointestinal reactions and 6 patients had menstrual abnormalities.

## Discussion

In this systematic review and meta-analysis, acupuncture combined with metformin was suggested to have a positive effect on pregnancy rate, ovulation rate, and HOMA-IR in patients with PCOS compared to metformin alone. Subgroup analysis showed that the causes of heterogeneity were related to diagnostic criteria and random methods.

These findings are consistent with previous systematic evaluation and RCT results. Acupuncture alone or in combination with Western medicine in treating PCOS infertility can improve pregnancy rate, ovulation rate, hormone level, ovarian function, insulin resistance, and obesity (15, 30–33). However, the research results of Wu et al. were contrary to this (34). This study reported that acupuncture was not effective at treating infertility in PCOS patients. From the perspective of trial design, this study broke the traditional “step-by-step” evidence-based medicine research mode; that is, literature studies, observational studies, and small RCTs were not performed before performing a large-sample, multicenter RCT (35). This differs from the studies we have included in this review. Among the specific therapeutic methods, the choice of needles, acupoints, stimulation intensity, qi generation, treatment frequency, and course of treatment all have an impact on the curative effect. Therefore, the different results of acupuncture efficacy may be related to the lack of uniformity and objectivity of the current relevant clinical research standards.

Secondly, through meta-analysis, it was found that different diagnostic criteria of PCOS can cause differences in heterogeneity, and the heterogeneity of Rotterdam diagnostic criteria (*I* ^2^ = 25%) was significantly lower than the overall heterogeneity (*I* ^2^ = 41%). Currently, the Rotterdam standard is generally accepted and internationally recognized. In this study, five articles (23, 24, 27–29) adopted this standard. Fifteen years later, based on the disease characteristics of Han women in China, epidemiological investigation and research on the Chinese PCOS population was conducted. The 2018 “Guidelines for Diagnosis and Treatment of Polycystic Ovarian Syndrome” in China were formulated; two of the included studies were based on this standard (21, 25). When comparing the two criteria, the Rotterdam diagnostic criteria have a wider range than China’s, but China’s criteria are more detailed. Chinese standards put forward the concept of “suspected PCOS” as the first step of diagnosis, with the second step being to confirm PCOS. As a result, the clinical studies using the Chinese standard included both patients with early suspected status and confirmed status. However, as the original data did not separate these two groups of patients, there was heterogeneity in the analysis. However, this improvement to the guidelines raises the bar for future health risk assessment, long-term clinical management, and pregnancy assistance strategies in patients who have not been fully diagnosed in the early stages of the condition.

Through different diagnostic criteria for pregnancy, our subgroup analysis found that the heterogeneity of pregnancy diagnosis by ultrasound was lower than that by laboratory indicators (hCG detection only in serum or urine). We think more about the diagnosis of pregnancy. Based on the last 3 years of studies retrieved from Clinical Trials.gov, the Chinese Clinical Trial Register and Prosper, in studies of PCOS and *in vitro* fertilization/intra-cytoplasmic sperm injection (IVF/ICSI) (36–39), ultrasound was widely used as the diagnostic standard for pregnancy. In the subgroup analysis, it was found that clinical pregnancy criteria were generally adopted relatively recently, indicating that Chinese clinical studies were closer to international studies.

### Quality of the evidence

In this review, we included only nine RCTs, most of which had small sample sizes. The quality of the evidence was low or very low. The main problems are risk of bias, imprecision, and inconsistency of the research results (**Table 2**).

### Limitations

The limitations of this systematic evaluation are as follows (1): the included intervention measures, such as acupuncture forms, acupoint selection, treatment frequency, and course of treatment, vary greatly, and further subgroup analysis cannot be carried out due to the limited number of studies, affecting the accuracy of the results; and (2) due to incomplete information about the authors of most of the studies, we were only able to contact four of the authors by email, and did not receive any replies. It can be seen that the author of the original study is not very positive about the return visit of the author of the systematic evaluation. It may be because the author doesnot commonly use this contact method, or the recognition and research significance of the systematic evaluation are not high within the industry, and the author is not confident in his own scheme.

## Conclusion

### Implications for practice

We cannot exclude clinically relevant differences in pregnancy rate, ovulation rate, LH/FSH, HOMA-IR, and FPG for acupuncture combined with metformin versus metformin. The pregnancy rate, ovulation rate and HOMA-IR of participants receiving acupuncture combined with metformin may have improved compared to metformin alone. Due to differences in pregnancy diagnostic criteria, we are not sure if this is effective in studies without a definitive B-ultrasound diagnosis. Due to the low quality of evidence and the limited number of RCTs available in this area, our ability to determine whether acupuncture combined with metformin is more effective at treating PCOS than metformin alone is limited.

#### Implications for research

It is hoped that acupuncture combined with metformin will improve the pregnancy rate of PCOS women.Further well-designed and well-performed randomized controlled trials are needed to definitively answer this question. Under uniform diagnostic criteria, a standard set of acupuncture points and stimulation methods should be considered, and the control group should receive the same metformin regimen as the acupuncture group.

## Data availability statement

The original contributions presented in the study are included in the article/supplementary material. Further inquiries can be directed to the corresponding author.

## Author contributions

YL and HYL conducted literature searches, evaluated study inclusion, and extracted data. XC and JLY analyzed data and drafted the manuscript. QLX’s assessment was incorporated into the study and cross-checked with XC. YL revised the language and the article. JW, XYZ, YMZ, CYL, MJW, and ZL conceived the study and revised the manuscript. All authors read and approved the final version of the manuscript.

## Funding

This study was supported by the National Natural Science Foundation of China (81774412 and 82105028) and the Sichuan Administration of Traditional Chinese Medicine (2021MS079).

## Conflict of interest

The authors declare that the research was conducted in the absence of any commercial or financial relationships that could be construed as a potential conflict of interest.

## Publisher’s note

All claims expressed in this article are solely those of the authors and do not necessarily represent those of their affiliated organizations, or those of the publisher, the editors and the reviewers. Any product that may be evaluated in this article, or claim that may be made by its manufacturer, is not guaranteed or endorsed by the publisher.

## References

[1] Zhang W Sun L Guo J Yu X Shi Y . [Family-based analysis of the adiponectin gene polymorphisms and polycystic ovary syndrome]. Zhonghua Fu Chan Ke Za Zhi (2014) 49:758–62. doi: 10.3760/cma.J.issn.0529-567x.2014.10.009 25537248

[2] Balen AH Morley LC Misso M Franks S Legro RS Wijeyaratne CN . The management of anovulatory infertility in women with polycystic ovary syndrome: an analysis of the evidence to support the development of global WHO guidance. Hum Reprod Update (2016) 22:687–708. doi: 10.1093/humupd/dmw025 27511809

[3] Lim SS Kakoly NS Tan JWJ Fitzgerald G Bahri Khomami M Joham AE . Metabolic syndrome in polycystic ovary syndrome: a systematic review, meta-analysis and meta-regression. Obes Rev (2019) 20(2):339–52. doi: 10.1111/obr.12762 30339316

[4] Dumesic DA Abbott DH . Implications of polycystic ovary syndrome on oocyte development. Semin Reprod Med (2008) 26(1):53–61. doi: 10.1055/s-2007-992925 18181083 PMC2655636

[5] Barbieri RL Makris A Randall RW Daniels G Kistner RW Ryan KJ . Insulin stimulates androgen accumulation in incubations of ovarian stroma obtained from women with hyperandrogenism. J Clin Endocrinol Metab (1986) 62(5):904–10. doi: 10.1210/jcem-62-5-904 3514651

[6] Badawy A Elnashar A . Treatment options for polycystic ovary syndrome. Int J Women’s Health (2011) 3:25–35. doi: 10.2147/IJWH.S11304 21339935 PMC3039006

[7] Cassar S Misso ML Hopkins WG . Insulin resistance in polycystic ovary syndrome: a systematic review and meta-analysis of euglycaemic-hyperinsulinaemic clamp studies. Hum Reprod (2016) 31(11):2619–31. doi: 10.1093/humrep/dew243 27907900

[8] Stepto NK Cassar S Joham AE Hutchison SK Harrison CL Goldstein RF . Women with polycystic ovary syndrome have intrinsic insulin resistance on euglycaemic-hyperinsulaemic clamp. Hum Reprod (2013) 28(3):777–84. doi: 10.1093/humrep/des463 23315061

[9] Lim SS Davies MJ Norman RJ Moran LJ . Overweight, obesity and central obesity in women with polycystic ovary syndrome: a systematic review and meta-analysis. Hum Reprod Update (2012) 18(6):618–37. doi: 10.1093/humupd/dms030 22767467

[10] Smith JF Eisenberg ML Millstein SG Nachtigall RD Shindel AW Wing H . The use of complementary and alternative fertility treatment in couples seeking fertility care: data from a prospective cohort in the united states. Fertil Steril (2010) 93(7):2169–74. doi: 10.1016/j.fertnstert.2010.02.054 PMC2860047 20338559

[11] Chen X Tang H Liang Y Wu P Xie L Ding Y . Acupuncture regulates the autophagy of ovarian granulosa cells in polycystic ovarian syndrome ovulation disorder by inhibiting the PI3K/AKT/mTOR pathway through LncMEG3. BioMed Pharmacother (2021) 144:112288. doi: 10.1016/j.biopha.2021.112288 34653763

[12] Jo J Lee YJ Lee H . Acupuncture for polycystic ovarian syndrome: A systematic review and meta-analysis. Medicine (2017) 96(23):e7066. doi: 10.1097/md.0000000000007066 28591042 PMC5466220

[13] Yu LQ Cao LY Shi Y Yuan YJ Jin X . A review of the effect and mechanism of acupuncture on polycystic ovary syndrome. Shanghai J Acupuncture Moxibustion (2015) 34:269–72. doi: 10.13460/j.issn.1005-0957. 2015. 03. 0269

[14] Shen Y Wang J . Acupuncture modulates the hypothalamic-pituitary-ovarian axis in the treatment of polycystic ovary syndrome: a research progress. Chin J Integrated Traditional Western Med (2022) 42:625–32. doi: 10.7661/cjim.20210913.365

[15] Johansson J Redman L Veldhuis PP Sazonova A Labrie F Holm G Johannsson G . Acupuncture for ovulation induction in polycystic ovary syndrome: a randomized controlled trial. Am J Physiol Endocrinol Metab (2013) 304(9):E934–943. doi: 10.1152/ajpendo.00039.2013 PMC4116535 23482444

[16] Liang F Koya D . Acupuncture: is it effective for treatment of insulin resistance? Diabetes Obes Metab (2010) 12:555–69. doi: 10.1111/j.1463-1326.2009.01192.x 20590731

[17] Shi Y Li L Zhou J Sun J Chen L Zhao J . Efficacy of electroacupuncture in regulating the imbalance of AMH and FSH to improve follicle development and hyperandrogenism in PCOS rats. BioMed Pharmacother (2019) 113:108687. doi: 10.1016/j.biopha.2019.108687 30851546

[18] Tarkun I Dikmen E Cetinarslan B Cantürk Z . Impact of treatment with metformin on adipokines in patients with polycystic ovary syndrome. Eur Cytokine Network (2010) 21(4):272–7. doi: 10.1684/ecn.2010.0217 21126943

[19] Page MJ Mckenzie JE Bossuyt PM Boutron I Hoffmann TC Mulrow CD . The PRISMA 2020 statement: an updated guideline for reporting systematic reviews. BMJ (2021) 372:n71. doi: 10.1371/journal.pmed.1003583 33782057 PMC8005924

[20] Higgins JPT Sterne JAC Savović J . A revised tool for assessing risk of bias in randomized trials. Cochrane Database Syst Rev (2016) 10(Supp 1):CD201601.

[21] Li YC Feng T Rong CF He MJ . Effect of ear acupuncture combined with metformin on insulin resistance in patients with phlegm-dampness polycystic ovary syndrome. J External Ther Traditional Chin Med (2021) 30:59–61.

[22] Wang JY Ma Y Du WN Wang BC Wu LQ . Clinical study of influence of metformin combined with acupuncture on glucolipid metabolism and adipokines in obese polycystic ovarian syndrome. China Modern Med (2020) 27:69–72.

[23] Li L Mo H Wen B Zhang J Li Y Chen WF . Clinical study of the acupuncture combined with metformin for infertility patients with obesity-type polycystic ovary syndrome. China J Traditional Chin Med Pharm (2014) 29:2115–9.

[24] Zhai ZY . Metformin combined with acupuncture for infertility of obese polycystic ovary syndrome. International Medicine and Health Guidance News (2017) 23:2403 –5. doi: 10.3760/cma.j.issn.1007-1245.2017.15.025

[25] Peng XY . Clinical observation of acupuncture combined with metformin in the treatment of polycystic ovarian infertility. Chin Community Doctors (2020) 36:121–2. doi: :10.3969/j.issn.1007-614x.2020.26.059

[26] Li SS . Metformin and auxiliary acupuncture in the treatment of obese women infertility with polycystic ovary syndrome for 75 cases. Chin Med Modern Distance Educ China (2015) 13(06):78–9. doi: 10.3969/j.issn.1672-2779.2015.06.039

[27] Tang J . Effects of acupuncture combined with metformin on sex hormone levels, glucose metabolism and pregnancy outcome in infertility patients with polycystic ovary syndromed. Zhejiang J Traditional Chin Med (2017) 52:568–9. doi: 10.13633/j.cnki.zjtcm.2017.08.014

[28] Zhang ZL . Effect of acupuncture combined with metformin on infertility of obese polycystic ovary syndrome. Med Forum (2016) 20:4670–1. doi: 10.19435/j.1672-1721.2016.33.036

[29] Liu YE Liao BD . Clinical observation of acupuncture plus metformin for infertile women with polycystic ovary syndrome. Shanghai J Acupuncture Moxibustion (2018) 37:1354–8. doi: 10.13460/j.issn.1005-0957.2018.12.1354

[30] Huang SQ Xu HY Xiong J Xiang J Hua FH . Efficacy of acupuncture for PCOS infertility: a systematic review. Chin J Evidence-Based Med (2021) 21:431–7. doi: 10.7507/1672-2531.202009166

[31] Wu D Wang XB Cong HF Liu SM Liang Y . Effects of acupuncture on the ovarian function in obese patients with polycystic ovary syndrome. World Chin Med (2020) 15:2482–5. doi: 10.3969/j.issn.1673-7202.2020.16.028

[32] Pastore LM Williams CD Jenkins J Patrie JT . True and sham acupuncture produced similar frequency of ovulation and improved LH to FSH ratios in women with polycystic ovary syndrome. J Clin Endocrinol Metab (2011) 96(10):3143–50. doi: 10.1210/jc.2011-1126 PMC3200239 21816787

[33] Shen LY Liang CM Yang WJ Pan L Li H Hu H . Acupuncture treatment of polycystic ovarian syndrome patients with abdominal obesity by regulating dai meridian: A randomized controlled clinical trial. Acupuncture Res (2018) 43(04):255–9. doi: 10.13702/j.1000-0607.170687 29888581

[34] Wu XK Stener-Victorin E Kuang HY Ma HL Gao JS Xie LZ . Effect of acupuncture and clomiphene in chinese women with polycystic ovary syndrome: a randomized clinical trial. JAMA (2017) 317(24):2502–14. doi: 10.1001/jama.2017.7217 PMC5815063 28655015

[35] Deng YY Gao JS Ma LH Wang R Chen XH Ma HX . Protocol optimization and quality control of large-scale acupuncture clinical trial for infertility. Zhongguo Zhen Jiu (2017) 37:541–4. doi: 10.13703/j.0255-2930.2017.05.023 29231617

[36] Hamdan M Dunselman G TC Li Cheong Y . The impact of endometrioma on IVF/ICSI outcomes: a systematic review and meta-analysis. Hum Reprod Update (2015) 21(6):809–25. doi: 10.1093/humupd/dmv035 26168799

[37] Xu J Yin MN Chen ZH Yang L Ye DS Sun L . Embryo retention significantly decreases clinical pregnancy rate and live birth rate: A matched retrospective cohort study. Fertil Steril (2020) 114(4):787–91. doi: 10.1016/j.fertnstert.2020.04.043 32771257

[38] Liu X Zhang W Xu Y Chu Y Wang X Li Q . Effect of vitamin d status on normal fertilization rate following *in vitro* fertilization. Reprod Biol Endocrinol (2019) 17(1):59. doi: 10.1186/s12958-019-0500-0 31319865 PMC6639905

[39] Lim CED Ng RWC Cheng NCL Zhang GS Chen H . Acupuncture for polycystic ovarian syndrome. Cochrane Database Syst Rev (2019) 7(7):Cd007689. doi: 10.1002/14651858.CD007689.pub4 31264709 PMC6603768

